# Morphological Analysis of the Flow–Volume Curve for Identifying Fixed Airflow Obstruction: A Scoping Review

**DOI:** 10.3390/arm94050061

**Published:** 2026-08-29

**Authors:** Alirio Bastidas-Goyes, Luis F. Giraldo-Cadavid, Eduardo Tuta-Quintero, Diana Diaz-Quijano, Daniel Botero-Rosas, Adriana Maldonado-Franco, Alejandra Vargas, Paula Rincón, Lina López, Juan S. Hernández, Juan Castro, Isabella Criado, Charbel Faizal-Gómez, David Jiménez, Ingrid Mora, Juan León

**Affiliations:** 1 Doctorate in Clinical Sciences, School of Medicine, Universidad de La Sabana, Campus del Puente del Común, Km 7 Autopista Norte de Bogotá, Chia 250001, Colombia; 2 School of Medicine, Universidad de La Sabana, Campus del Puente del Común, Km 7 Autopista Norte de Bogotá, Chia 250001, Colombia; luisf.giraldo@unisabana.edu.co (L.F.G.-C.); diana.diaz1@unisabana.edu.co (D.D.-Q.); daniel.botero@unisabana.edu.co (D.B.-R.); adriana.maldonado@unisabana.edu.co (A.M.-F.); alejandravacu@unisabana.edu.co (A.V.); paularinri@unisabana.edu.co (P.R.); linalonu@unisabana.edu.co (L.L.); juanhepu@unisabana.edu.co (J.S.H.); juancascor@unisabana.edu.co (J.C.); isabellacrqu@unisabana.edu.co (I.C.); charbelfago@unisabana.edu.co (C.F.-G.); davidjimo@unisabana.edu.co (D.J.); ingridmove@unisabana.edu.co (I.M.); juanlebe@unisabana.edu.co (J.L.); 3 Department of Internal Medicine and Epidemiology, School of Medicine, Universidad de La Sabana, Chia 250001, Colombia; eduardotuqu@unisabana.edu.co

**Keywords:** chronic obstructive pulmonary disease, spirometry, pulmonary function tests, airway obstruction, curve fitting

## Abstract

**Highlights:**

**What are the main findings?**

- This scoping review identified 24 studies evaluating morphological analysis of the expiratory flow–volume curve for the identification and characterization of fixed airflow obstruction, with most evidence focused on handcrafted descriptors such as concavity, expiratory slope, and area-derived metrics, which consistently showed strong associations with airflow obstruction and related physiological and structural abnormalities, including emphysema, hyperinflation, and disease severity.
- A smaller but growing body of evidence demonstrated that mathematical modeling and machine-learning approaches applied to flow–volume curve morphology achieved high diagnostic accuracy and showed promise for the automated identification of fixed airflow obstruction, physiological characterization, prognostic assessment, and risk stratification.

**What are the implications of the main findings?**

- Morphological analysis of the expiratory flow–volume curve may provide clinically meaningful information beyond conventional spirometric indices, particularly for early detection of airflow limitation, phenotyping, and assessment of disease severity, while remaining compatible with routine spirometry.
- Future research should prioritize prospective multicenter validation, standardized definitions of morphological metrics, and external validation of artificial intelligence models to facilitate integration of these approaches into clinical practice and pulmonary function reporting.

**Abstract:**

**Background/Objective:** The morphology of the flow–volume curve has emerged as a potential source of additional functional information by enabling the analysis of concavity, slope-based metrics, area-derived measures, and mathematical models extracted from the expiratory tracing. Therefore, the aim of this study was to map and describe the available evidence on morphological analysis methods of the expiratory flow–volume curve for the identification and characterization of fixed airflow obstruction. **Methods:** A scoping review was conducted following the methodological frameworks proposed by Arksey and O’Malley, Levac et al., the Joanna Briggs Institute, and the PRISMA Extension for Scoping Reviews (PRISMA-ScR). Studies published between 1 January 1990, and 31 December 2025, that evaluated morphological flow–volume curve metrics for the diagnosis or characterization of Chronic Obstructive Pulmonary Disease (COPD) were included, without language restrictions. Searches were performed in PubMed/MEDLINE, Embase, Scopus, Web of Science, IEEE Xplore, OpenGrey, and Google Scholar. Study selection was conducted by independent reviewers, with disagreements resolved by consensus, and data extraction was performed using a standardized form. Results were synthesized narratively and organized according to metric families. **Results:** The search identified 13,577 records; after duplicate removal and screening, 24 studies met the inclusion criteria. The evidence included observational studies, diagnostic validation studies, longitudinal cohorts, and methodological modeling studies. Identified metrics were grouped into four main categories: concavity and geometric indices of the flow–volume curve, expiratory slope and flow-decay metrics, area-based or volumetric-derived measures, and mathematical or computational models applied to the tracing. Concavity indices, the β-angle, slope-ratio, Peak Index, and the D parameter showed consistent associations with airflow obstruction, emphysema, small airway disease, or functional impairment. Expiratory slope metrics and Flow Decay demonstrated high diagnostic performance in several studies, whereas area-based measures such as AEX, AEX-FV, AreaFE%, and AUC3/AT3 integrated the overall loss of expiratory flow during forced expiration. Mathematical and computational models suggested that the complete shape of the curve contains additional diagnostic and prognostic information, although methodological variability and the need for external validation remain important limitations. **Conclusions:** Morphological analysis of the flow–volume curve represents a promising approach to complement conventional spirometry in the identification and characterization of fixed airflow obstruction. These metrics may provide additional information regarding airflow limitation, non-uniform lung emptying, emphysema, small airway disease, hyperinflation, and clinically relevant outcomes.

## 1. Introduction

Chronic obstructive pulmonary disease (COPD) is a heterogeneous respiratory condition characterized by chronic symptoms such as dyspnea, cough, and sputum production, associated with persistent abnormalities of the airways and/or lung parenchyma that result in progressive airflow limitation [1,2]. Its global prevalence among adults older than 40 years is estimated at approximately 10%, affecting hundreds of millions of individuals worldwide, and the number of cases is projected to reach nearly 600 million by 2050, particularly in low- and middle-income countries [3,4]. Furthermore, the global economic burden attributable to COPD is expected to exceed INT$4.326 trillion between 2020 and 2050 [5]. In Colombia, the estimated spirometric prevalence among adults older than 40 years is 8.9% [6].

Currently, the functional diagnosis of COPD is based on demonstrating a post-bronchodilator FEV_1_/FVC ratio below 0.70 [7]. However, this approach has important limitations. Small airway disease may progress substantially before detectable abnormalities appear in conventional spirometric indices, thereby reducing diagnostic sensitivity in the early stages of the disease [8,9,10,11,12]. In addition, spirometry requires prolonged and technically demanding expiratory maneuvers, which may affect reproducibility and make testing difficult in elderly patients or those with severe dyspnea [9,10].

Given these limitations, the morphology of the flow–volume curve (FVCu) has emerged as a potential source of complementary physiological information. Several studies have demonstrated that the shape of the expiratory limb is associated with structural and functional abnormalities characteristic of COPD, particularly emphysema and small airway disease [13,14]. Based on this premise, multiple metrics have been developed to extract additional diagnostic information from the geometry of the curve.

Among the most extensively studied approaches are concavity indices, which quantify deformation of the descending limb of the flow–volume curve and have shown correlations with the extent of emphysema and other structural manifestations of the disease [13,14,15,16,17]. Similarly, slope-based metrics have been proposed, such as Flow Decay, which estimates dynamic airway resistance from the exponential decline in expiratory flow [15], as well as slope analyses derived from peak expiratory flow or specific segments of the forced vital capacity. These measures have demonstrated diagnostic accuracy comparable to that achieved with traditional spirometric criteria [16].

Another strategy involves area-based metrics, such as the Area under the Expiratory Curve (AEX) and its derived variants, which integrate the overall behavior of forced expiration and have shown utility in identifying ventilatory abnormalities even in incomplete spirometric maneuvers [18,19,20]. More recently, advances in mathematical modeling and machine learning have enabled the analysis of the flow–volume curve as a complex signal, leading to the development of algorithms capable of discriminating between individuals with and without COPD and improving functional characterization through automated pattern recognition [8].

Collectively, these approaches aim to complement conventional spirometric indices by leveraging the information contained within the geometry of the flow–volume curve. Nevertheless, the available evidence remains heterogeneous, with substantial differences in the methodologies employed, the reference standards used, and the reported performance metrics [19,20]. This variability hinders the integration of findings and limits their clinical applicability. Therefore, a comprehensive synthesis of the existing evidence is needed to better understand the different morphological analysis strategies and to establish a foundation for future research focused on the validation and standardization of more sensitive spirometric measures for the early identification and characterization of fixed airflow obstruction.

## 2. Methods

A scoping review was conducted to map and describe the available evidence on morphological analysis methods of the expiratory flow–volume curve for the identification and characterization of fixed airflow obstruction. The review was performed in accordance with the methodological framework proposed by Arksey and O’Malley, subsequently refined by Levac et al., as well as the recommendations outlined in the Joanna Briggs Institute (JBI) Reviewers’ Manual for Scoping Reviews [21,22,23]. Reporting of the review findings followed the Preferred Reporting Items for Systematic Reviews and Meta-Analyses Extension for Scoping Reviews (PRISMA-ScR) guidelines (Supplementary Table S1) [24].

The review comprised the following stages: (a) identification of the research question; (b) identification of relevant studies; (c) selection of eligible studies; (d) data extraction; and (e) synthesis and presentation of results. Upon completion of the study selection process, the protocol was registered on the Open Science Framework (OSF) platform and accessed on 26 May 2026: https://osf.io/wz5pd/overview.

### 2.1. Eligibility Criteria

Studies published between 1 January 1990, and 31 December 2025, that evaluated morphological analysis methods of the expiratory flow–volume curve for the diagnosis or characterization of COPD were eligible for inclusion. Eligible study designs comprised observational studies (cross-sectional, cohort, and case–control studies), clinical trials, and literature reviews. No language restrictions were applied; therefore, studies published in languages other than English were eligible for inclusion provided that sufficient information was available to assess their eligibility and extract the relevant data.

Studies were excluded if they (1) did not evaluate morphological characteristics or shape-based parameters of the expiratory flow–volume curve; (2) did not address COPD diagnosis or characterization; (3) evaluated artificial intelligence–based approaches without an explicit morphological analysis of the expiratory flow–volume curve; (4) were research protocols, case reports, or case series; or (5) did not provide sufficient information to assess the study methods or relevant outcomes. Studies were also excluded when the full text could not be obtained after the available retrieval strategies. When multiple publications reported data from the same study population, the publication providing the most complete and relevant information was retained.

### 2.2. Information Sources and Search Strategy

The search was conducted in three stages: an initial exploratory search to identify relevant terms, a systematic search of electronic databases and gray literature sources, and a manual search of the reference lists of included articles.

The electronic databases searched were PubMed/MEDLINE and Embase (biomedical databases), Scopus and Web of Science (multidisciplinary databases), and IEEE Xplore (engineering database). Gray literature was explored through OpenGrey and Google Scholar.

The search strategy was initially structured using the PICO framework, considering individuals older than 40 years with COPD risk factors as the population; any technique or model for morphological analysis of the expiratory flow–volume curve as the intervention; conventional spirometry, clinical assessment, or radiological findings as reference comparators; and COPD diagnosis as the outcome. Given the exploratory nature of the review, the Population–Concept–Context (PCC) framework recommended for scoping reviews was also considered [23].

Search terms included Medical Subject Headings (MeSH) and keywords related to risk factors, flow–volume curve modeling, and COPD diagnosis, including (Supplementary Table S2): “Risk Factors,” “Tobacco Use,” “Biomass Exposure,” “Air Pollution,” “Mathematical Model,” “Morphology,” “Concavity,” “Area Under Curve,” “Slope,” “Curve Fitting,” “Flow–volume Loop,” “Spirometry,” “Expiratory Flow,” “Pulmonary Disease, Chronic Obstructive,” “Airflow Obstruction,” “Early Diagnosis,” “Diagnostic Accuracy,” “Sensitivity and Specificity,” “Reproducibility,” and “Reliability.”

### 2.3. Study Selection, Data Extraction, and Synthesis

Search results were imported into Rayyan software for duplicate removal and screening. Before formal screening, a pilot calibration exercise was conducted using a random sample of 25 titles and abstracts to ensure consistent application of the eligibility criteria. Screening commenced once inter-rater agreement exceeded 75%, after which the prevalence-adjusted bias-adjusted kappa (PABAK) coefficient was calculated.

Study selection was independently performed by two reviewers, with disagreements resolved through discussion with a senior investigator. Potentially eligible studies subsequently underwent full-text review. Reasons for exclusion were documented and reported in the PRISMA-ScR flow diagram.

Data extraction was independently conducted by four reviewers using a standardized data collection form. Extracted variables included authors, year of publication, study design, type of morphological metric evaluated, reference standard used, reported diagnostic performance, and aspects related to reproducibility or technical feasibility. Morphological metrics were classified according to the primary characteristic of the expiratory flow–volume curve that they quantified. Concavity and geometric indices described the shape and degree of inward curvature of the expiratory limb and included central or peripheral concavity indices, β angle, slope-ratio, obstructive index, peak index, and related geometric descriptors. Expiratory slope and flow-decay metrics quantified the rate at which expiratory flow decreased as lung volume fell, including the PEF–FVC slope, flow decay index, and spirometric time constant. Area-derived measures quantified the area under the expiratory flow–volume curve or the proportion of the curve area within predefined volume or flow segments, including AEX, AEX-FV, obstruction area, AreaFE%, and AUC3/AT3. These handcrafted descriptors were distinguished from mathematical and machine-learning approaches, which used fitted mathematical functions, polynomial representations, or data-driven models applied to raw or parameterized flow–volume curves. This classification was used to organize the included studies according to the principal morphological feature evaluated. Results were synthesized narratively and organized into summary tables.

## 3. Results

### 3.1. Study Selection and Characteristics

**Figure 1:**
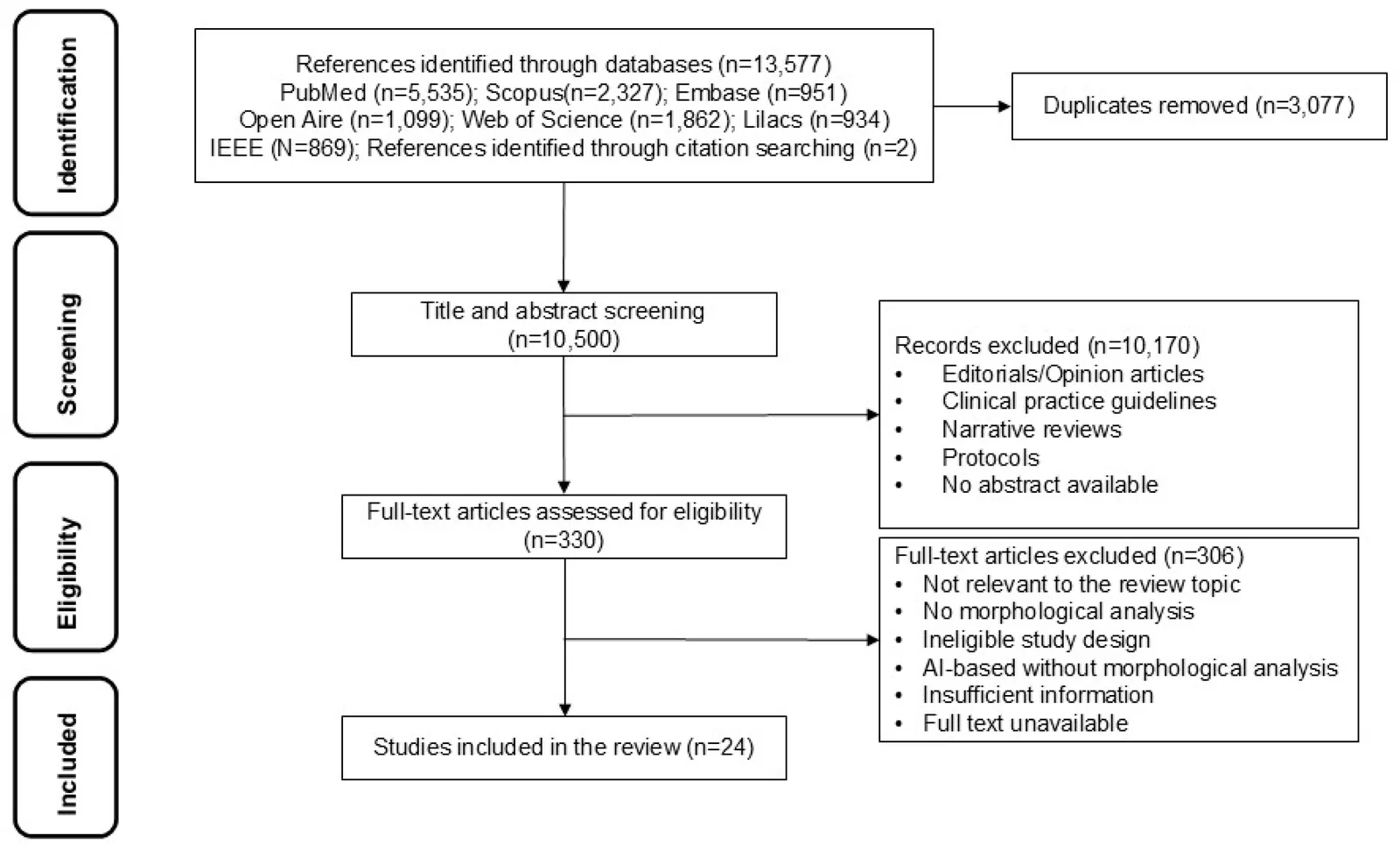
PRISMA flow diagram.

The search identified 13,577 records, of which 10,500 remained after duplicate removal and were screened by title and abstract (Figure 1). Of these, 330 articles underwent full-text assessment, and 316 were excluded. Ultimately, 24 studies met the inclusion criteria [9,13,15,16,18,20,25,26,27,28,29,30,31,32,33,34,35,36,37,38,39,40,41,42]. Inter-rater agreement improved from poor concordance during the initial calibration phase (κ = 0.094) to substantial agreement during the final screening stage (κ = 0.746; 95% CI, 0.476–1.000), with an observed agreement of 88%.

The characteristics of the included studies are summarized in Table 1 and Table 2. The studies encompassed cross-sectional, cohort, longitudinal, and clinical respiratory laboratory investigations, with sample sizes ranging from 33 to approximately 400,000 participants. Several studies used population-based or multicenter cohorts, whereas others evaluated patients referred for pulmonary function testing or selected COPD populations. Most studies assessed handcrafted morphological descriptors, while a smaller group evaluated mathematical representations or machine-learning models of the flow–volume curve. According to the primary morphological feature assessed, studies were grouped into (1) concavity and geometric indices, (2) expiratory slope and flow-decay metrics, and (3) area-derived measures. Mathematical and machine-learning approaches were considered separately (Table 1 and Table 2).

### 3.2. Concavity and Geometric Indices

Seven studies evaluated geometric characteristics or concavity of the expiratory flow–volume curve (Table 1). These studies included cross-sectional populations, clinical cohorts, and longitudinal studies, with sample sizes ranging from 34 to 18,938 participants.

In a large population and clinical cohort of 18,938 participants, Wang et al. evaluated the β angle and central and peripheral concavity indices for classification of spirometric patterns. The β angle demonstrated excellent discriminatory performance, with an AUROC of 0.97 using a cutoff of 163°, and an overall classification accuracy of 0.919. These findings support the ability of geometric descriptors to distinguish abnormal ventilatory patterns using information contained in the shape of the expiratory limb beyond conventional FEV_1_ and FVC measurements [28].

Johns et al., in a community-based sample of 890 adults, demonstrated strong correlations between central and peripheral concavity indices and conventional markers of airflow obstruction, particularly FEV_1_/FVC and FEF_25–75_% (r = −0.70 to −0.79), with high reproducibility (ICC approximately 0.96) [25]. In a smaller study of patients with mild COPD, the slope-ratio showed an altered volume-dependent pattern despite preserved FEV_1_/FVC, suggesting potential sensitivity to early abnormalities not captured by conventional spirometric thresholds [27].

**Table 2:** Mathematical and machine-learning models of the flow–volume curve.

Study	Population/Setting (N)	Model/Metric	Clinical Target/Comparator	Key Finding
Bhatt et al. [9]	Multicenter cohort of smokers/former smokers at risk for COPD (8307)	D parameter	CT structural disease; discordant mild airflow obstruction	r(FEV_1_/FVC) = −0.83; AUC 0.83 for emphysema > 5%; AUC 0.76 for fSAD; adjusted HR 3.22
Maldonado-Franco et al. [38]	Outpatient clinical spirometry (695)	Polynomial/exponential coefficients + supervised learning	COPD vs. non-COPD classification	Neural network AUC 0.929; Se 0.882; Sp 0.943; PPV 0.833; NPV 0.962
Hebbink et al. [39]	Healthy subjects and pulmonary disease (58)	Legendre polynomials	Relationship with Tiffeneau-Pinelli index	R^2^ 0.92 for forced curves; R^2^ 0.59 for tidal curves
Gadgil et al. [40]	Population-based cohort with longitudinal records (~400,000)	Time-series transformer on raw flow–volume curves	COPD risk, mortality, exacerbations	AUC 0.828 for COPD risk; 0.937 for mortality; 0.851 for exacerbations

AUC, area under the receiver operating characteristic curve; COPD, chronic obstructive pulmonary disease; CT, computed tomography; FEV_1_, forced expiratory volume in one second; FVC, forced vital capacity; fSAD, functional small-airway disease; HR, hazard ratio; NPV, negative predictive value; PPV, positive predictive value; Se, sensitivity; Sp, specificity.

Longitudinal and disease-specific studies further supported the clinical relevance of these geometric descriptors. Tan et al. evaluated 852 participants over an 8-year follow-up period and found that central concavity was associated with incident COPD, with an AUROC of 0.76, sensitivity of 0.70, specificity of 0.79, and negative predictive value of 0.99 [26]. In 133 patients with stable obstructive lung disease, the obstructive index was associated with emphysema burden assessed by CT, with an AUROC of 0.819 and a correlation with low-attenuation volume percentage of 0.56 [13]. Similarly, the lung size-adjusted peak index, evaluated in 8390 smokers and former smokers, was associated with lung function impairment, structural lung abnormalities, and longitudinal outcomes, including FEV_1_ decline; correlations with FEV_1_/FVC and predicted FEV_1_ were −0.73 and −0.67, respectively, with high reproducibility (ICC = 0.923) [29]. Collectively, these findings indicate that concavity and geometric descriptors may provide information related to airflow obstruction, early disease, emphysema, and longitudinal functional impairment.

### 3.3. Expiratory Slope and Flow-Decay Metrics

Four studies evaluated the rate of flow decline during expiration or related time-dependent characteristics of the flow–volume curve. In 765 patients presenting with chronic respiratory symptoms, Moreno-Giraldo et al. evaluated the PEF–FVC slope against post-bronchodilator FEV_1_/FVC < 0.70 as the diagnostic reference standard. A cutoff of >−1.76 yielded an AUROC of 0.98, sensitivity of 0.94, specificity of 0.94, and ICC of 0.83, indicating excellent discrimination and good reproducibility [16].

In a pulmonary function laboratory cohort of 125 participants, the flow-decay index differentiated healthy individuals from patients with reversible obstruction and from those with obstruction accompanied by hyperinflation or air trapping. The reported AUROCs ranged from 0.95 to 0.98, with a coefficient of variation of 9.8 ± 3.4%, supporting both diagnostic discrimination and measurement reproducibility [15].

Beyond diagnostic classification, the spirometric time constant showed prognostic and structural associations in a population-based cohort and an at-risk smoking population comprising 8301 participants. The metric was associated with COPD progression, emphysema, functional small-airway disease, and mortality, suggesting that flow-decay characteristics may capture clinically relevant pathophysiological information beyond conventional spirometric indices [30]. In a large occupational surveillance cohort of 10,810 participants, longitudinal transitions toward a low-FVC pattern were also associated with changes in ventilatory physiology, with the low-FVC phenotype defined by FVC below the lower limit of normal and FEV_1_/FVC above the lower limit of normal [31].

### 3.4. Area-Derived Flow–Volume Curve Metrics

Area-derived measures constituted another major group of handcrafted descriptors and were evaluated across diagnostic, physiological, and prognostic outcomes. These studies included clinical laboratory cohorts, COPD populations, primary-care populations, and challenge or bronchodilation testing cohorts.

In one of the largest pulmonary function laboratory studies, Ioachimescu et al. evaluated 15,308 participants and examined the actual area under the expiratory curve (AEX) for discrimination of normal, obstructive, restrictive, and mixed ventilatory patterns. AEX demonstrated excellent discriminatory performance, with AUROCs ranging from 0.97 to 0.99, and was also associated with measures of static lung volumes, including inspiratory capacity, IC/TLC, and RV/TLC [42]. In the same-sized cohort, a simplified AEX4 metric demonstrated AUROCs ranging from 0.96 to 0.99 and correlated strongly with actual AEX (r = 0.95–0.99), suggesting that simplified area-based measurements may retain much of the diagnostic information of the complete curve area [20].

Other studies evaluated area-derived measures in specific COPD-related outcomes. In 540 patients with respiratory disease, AEX-FV showed good discrimination of bronchial obstruction, with sensitivity ranging from 0.824 to 0.916 and specificity from 0.800 to 0.933 [33]. Among 319 patients with COPD, AreaFE% was strongly associated with severe hyperinflation, defined by RV/TLC > 60% or IC/TLC < 25%, with an AUROC of 0.91 and correlations of −0.78 with RV/TLC and 0.80 with IC/TLC [35]. In 81 patients with COPD and severe dyspnea, the same metric was associated with severe exacerbations, with an AUROC of 0.88, sensitivity of 0.818, specificity of 0.828, and negative predictive value of 0.96 [36].

Additional area-based approaches showed utility for identifying airflow limitation and bronchial hyperresponsiveness. AEX-derived measures were associated with bronchodilator response and bronchial hyperresponsiveness in challenge testing, while obstruction-area measures were associated with hyperinflation and exercise capacity [34]. In a hospital spirometry database of 500 participants, AUC3/AT3 correlated strongly with airflow limitation defined by FEV_1_/FVC (r = 0.88), with sensitivity of 0.865 and specificity of 0.859 [18]. In a primary-care population of 767 participants, FEV_1_/FEV_6_ demonstrated excellent discrimination for COPD compared with FEV_1_/FVC < 0.70, with an AUROC of 0.98, sensitivity of 0.969, and specificity of 0.988 [37].

Overall, area-derived metrics demonstrated consistently high diagnostic performance across different applications and were also associated with hyperinflation, exercise limitation, bronchial hyperresponsiveness, and exacerbation risk. These findings suggest that the integrated area of the expiratory curve may capture complementary information regarding airflow limitation and ventilatory mechanics.

### 3.5. Mathematical Modeling and Machine-Learning Approaches

Four studies evaluated mathematical representations or machine-learning models applied to raw or parameterized flow–volume curves (Table 2). These approaches ranged from polynomial and exponential curve fitting to Legendre polynomial representations and deep learning models.

Bhatt et al. evaluated the D parameter derived from the expiratory flow–volume curve in 8307 smokers and former smokers at risk for COPD. The parameter correlated strongly with FEV_1_/FVC (r = −0.83) and demonstrated discriminatory ability for emphysema (AUROC 0.83) and functional small-airway disease (AUROC 0.76). It was also associated with subsequent mortality risk, with an adjusted hazard ratio of 3.22, suggesting potential prognostic value beyond conventional spirometric classification [9].

In an outpatient cohort of 695 participants, polynomial and exponential coefficients derived from the flow–volume curve were used in supervised learning models for COPD classification. The neural-network model achieved an AUROC of 0.929, sensitivity of 0.882, specificity of 0.943, PPV of 0.833, and NPV of 0.962 [38]. Earlier work using Legendre polynomial representations in 58 individuals demonstrated that forced expiratory curves could be accurately reconstructed, with R^2^ = 0.92 for forced curves [39].

Gadgil et al. applied a transformer-based time-series model directly to raw flow–volume curves in a population-based cohort containing approximately 400,000 longitudinal records. The model demonstrated strong discrimination for COPD risk (AUROC 0.828), mortality (AUROC 0.937), and exacerbations (AUROC 0.851), highlighting the potential of automated curve analysis for both diagnostic and prognostic risk stratification [40].

### 3.6. Diagnostic, Prognostic, and Technical/Reproducibility Outcomes

Across the included studies, morphological analysis of the expiratory flow–volume curve was evaluated for three main types of outcomes: diagnostic discrimination, characterization of structural and physiological abnormalities, and prognostic risk stratification [9,13,15,16,18,20,25,26,27,28,29,30,31,32,33,34,35,36,37,38,39,40,41,42]. Diagnostic studies primarily compared morphological metrics with conventional spirometric thresholds or ventilatory pattern classification and generally reported high discrimination [15,16,18,20,25,26,27,28,33,37,38,42]. Other studies examined associations with CT-defined emphysema, hyperinflation, small-airway disease, exercise limitation, or longitudinal decline [9,13,28,29,30,31,34,35,36]. Prognostic analyses evaluated exacerbations, COPD progression, and mortality, with several morphological metrics demonstrating associations with clinically relevant outcomes [9,30,31,36,40].

Reproducibility was explicitly assessed in several studies and was generally favorable [15,16,25,29,33]. Reported ICC values ranged from approximately 0.83 to 0.96 for selected handcrafted descriptors, while area-based measures and concavity indices demonstrated particularly high repeatability [16,25,29]. However, heterogeneity in study populations, reference standards, definitions of morphological variables, and analytical methods limited direct comparison of diagnostic and prognostic performance across studies [9,13,15,16,18,20,25,26,27,28,29,30,31,32,33,34,35,36,37,38,39,40,41,42].

## 4. Discussion

This review identified that morphological analysis of the expiratory flow–volume curve in COPD has been developed primarily through four approaches: concavity and geometric indices, segmental slope metrics, area-based measures, and mathematical or machine learning models. Collectively, these methods seek to extract additional information from the shape of the expiratory limb, complementing the assessment provided by conventional spirometric parameters. The reviewed studies suggest that flow–volume curve morphology may provide additional insights into fixed airflow obstruction and its associated physiological, structural, and prognostic features in patients with COPD, including abnormalities that are not always fully captured by FEV_1_ or the FEV_1_/FVC ratio.

Concavity of the descending limb represents one of the most characteristic morphological features of airflow obstruction. From a pathophysiological perspective, this deformation has been associated with heterogeneous lung emptying, premature airway closure, and expiratory flow limitation through mechanisms such as the choke point phenomenon and wave-speed limitation [43,44]. Because small airway disease is a central component of COPD pathophysiology [45], it is plausible that these abnormalities manifest as alterations in curve morphology.

The included studies support this interpretation. Johns et al. demonstrated that central and peripheral concavity indices identified individuals with functional abnormalities that were not detected by conventional parameters and showed greater sensitivity than FEF_25–75_% for identifying early small airway involvement [25]. Similarly, Dominelli et al. found that the slope-ratio index detected characteristic curve deformations even in patients with mild COPD and relatively preserved FEV_1_ values, suggesting utility for the early detection of functional impairment [27]. Wang et al. reported that the β-angle achieved outstanding discriminatory performance for distinguishing obstructive from normal patterns (AUC = 0.970), further supporting the diagnostic potential of geometric descriptors [28]. In addition, Sarkar highlighted the clinical utility of morphological assessment for differentiating peripheral airway obstruction, upper airway obstruction, and restrictive disorders [46].

Beyond functional obstruction, concavity also appears to reflect relevant structural abnormalities. Mochizuki et al. demonstrated that the obstructive index was independently associated with the percentage of low-attenuation areas on computed tomography, a marker of emphysema, but not with bronchial wall thickness, suggesting a closer relationship with parenchymal destruction than with airway remodeling [13]. Likewise, Tan et al. found that greater central and peripheral concavity was associated with respiratory symptoms and a higher future incidence of COPD during follow-up, although its discriminatory performance was lower than that of the post-bronchodilator FEV_1_/FVC ratio [26]. Bhatt et al. extended this concept through the Peak Index, which was associated with emphysema and small airway disease [29]. Taken together, these findings suggest that concavity may represent an integrated manifestation of both functional and structural abnormalities.

Slope-derived metrics constitute a second major category of morphological measures. These indices quantify the rate of flow deceleration across different segments of the expiratory curve, providing a dynamic representation of forced expiration. Their pathophysiological rationale is based on the fact that small airway disease alters the rate of lung emptying and modifies the slope of expiratory flow decline [45].

Among the reviewed studies, Moreno-Giraldo et al. showed that the slope between peak expiratory flow and forced vital capacity (PEF–FVC slope) and the slope measured at 50% of FVC achieved AUC values of 0.98 and 0.99, respectively, for identifying airflow obstruction defined by FEV_1_/FVC <0.70 [16]. These findings suggest that simple geometric measures may approach the diagnostic performance of traditional spirometric parameters. Oh et al. proposed the Flow Decay index as an estimator of dynamic airway resistance, reporting high diagnostic performance for detecting reversible airflow obstruction, hyperinflation, and air trapping [15]. Complementarily, Bodduluri et al. introduced the spirometric time constant (Tc), demonstrating that it identified individuals at increased risk of exacerbations, emphysema, small airway disease, and mortality, even when conventional criteria remained within normal limits [30].

Nevertheless, the available evidence indicates that slope-based metrics do not constitute a homogeneous category. Their clinical significance depends on the segment analyzed, the calculation method employed, and the outcome being assessed. Weber et al., for example, used longitudinal modeling to investigate transitions between respiratory functional patterns, providing insights into the temporal evolution of pulmonary function rather than the diagnosis of airflow obstruction [31]. Therefore, these metrics should be interpreted according to their physiological basis and intended clinical application.

Area-based metrics represent the third major analytical approach. The area under the expiratory flow–volume curve (AEX) integrates the overall behavior of forced expiration and may capture functional abnormalities distributed throughout the entire tracing. This characteristic is particularly attractive in COPD, where airflow limitation may affect multiple portions of the curve.

Zapletal et al. demonstrated that a 20% reduction in AEX detected bronchoprovocation-induced functional abnormalities in all studied patients, substantially outperforming FEV_1_ sensitivity [32]. Subsequently, Ioachimescu and Stoller showed that different geometric approximations of AEX were highly correlated with the actual measurement and accurately discriminated among different ventilatory patterns [18,19]. Similarly, Desyatskova et al. reported a diagnostic specificity of 88.6% for AEX-FV in the detection of bronchial obstruction [33].

However, area-based metrics are heterogeneous and likely reflect different physiological dimensions. Lee et al. found associations between Ao/AR, Ao/PEF, and functional capacity measured by the six-minute walk test [34]. Das et al. demonstrated that AreaFE% identified severe hyperinflation with a diagnostic accuracy approaching 80%, whereas Satici et al. observed an independent association between reduced AreaFE% and severe exacerbations [36]. Other metrics, such as AUC3/AT3, were developed primarily to address technical limitations associated with incomplete spirometric maneuvers and demonstrated strong agreement with the FEV_1_/FVC ratio [35]. Likewise, Wang et al. validated the FEV_1_/FEV_6_ ratio as a practical alternative to FEV_1_/FVC in primary care settings [37]. Nevertheless, some studies reported more modest diagnostic performance, indicating that not all area-based metrics possess the same clinical potential [16].

Finally, mathematical models and machine learning techniques represent the most recent and sophisticated analytical approach. These methods are based on the premise that the entire curve contains distributed functional information that can either be summarized through mathematical parameters or processed using advanced algorithms. This perspective aligns with classical physiological models that describe expiratory flow limitation as a complex phenomenon determined by multiple mechanical properties [43,47].

Bhatt et al. demonstrated that the D parameter, derived from the volume-time curve, correlated with disease severity, was associated with emphysema and small airway disease, and identified individuals with structural abnormalities not recognized by conventional criteria [9]. Maldonado-Franco et al. used coefficients derived from polynomial and exponential curve fitting as inputs for artificial neural networks, achieving diagnostic accuracies exceeding 90% [38]. Hebbink et al. showed that Legendre polynomials could adequately describe the relationship between tidal and forced respiratory curves, although clinical applications remain limited [39]. More recently, Gadgil et al. applied Transformer-based models to nearly 400,000 participants and achieved strong predictive performance for mortality and exacerbations. However, the physiological interpretability of these algorithms remains limited and requires further validation before routine clinical implementation [40].

From a clinical perspective, morphological analysis of the expiratory flow–volume curve may provide complementary information beyond conventional spirometric indices, potentially contributing to the early detection, structural characterization, functional assessment, and prognostic stratification of COPD, particularly regarding small-airway dysfunction and emphysema [13,14,25,26,36]. However, substantial heterogeneity in calculation methods, thresholds, populations, and outcomes currently limits clinical implementation [19,38]. Therefore, these metrics should be considered complementary and exploratory rather than substitutes for conventional spirometric criteria, and the current evidence does not support changes to COPD diagnostic or management guidelines. Prospective, multicenter studies with external validation are needed to establish standardized metrics and define their incremental clinical value [19,38].

### Limitations

The available evidence was substantially heterogeneous in terms of study designs, populations, clinical settings, reference standards, and flow–volume curve morphological metrics, limiting direct comparability and generalizability of the findings. In addition, the lack of standardized definitions and calculation methods for morphological parameters, including concavity, slopes, areas, and mathematical models, precludes the identification of a single metric that can currently be recommended for clinical practice. Most studies were observational or diagnostic validation studies, limiting causal inference and the assessment of the impact of these metrics on clinical decision-making. The frequent use of conventional spirometric indices, particularly the FEV_1_/FVC ratio, as reference standards may also favor metrics derived from the same flow–volume curve and limit their ability to identify abnormalities preceding conventional diagnostic criteria. Furthermore, the reproducibility and external validity of several metrics remain uncertain, as many findings were derived from single cohorts or specific populations. Finally, mathematical and machine-learning approaches remain exploratory and require greater physiological interpretability, transparency, and independent external validation before routine clinical implementation. Therefore, flow–volume curve morphological metrics should currently be considered complementary tools rather than replacements for conventional spirometric criteria.

## 5. Conclusions

The available evidence indicates that morphological metrics derived from the expiratory flow–volume curve may provide complementary information beyond conventional spirometric indices. Concavity indices, expiratory slope measures, area-based metrics, and mathematical or machine-learning approaches have shown associations with non-spirometric markers of disease, including emphysema, hyperinflation, small-airway disease, functional impairment, exacerbations, and mortality. Although several metrics demonstrated strong correlations with airflow obstruction defined by conventional spirometric criteria, current evidence does not support their use as replacements for spirometry in the diagnosis of COPD. Methodological heterogeneity, differences in reference standards, and limited external validation currently restrict their clinical applicability.

## Figures and Tables

**Table 1:** Handcrafted flow–volume curve descriptors.

Study	Population/Setting (N)	Metric	Clinical Target/Comparator	Key Finding
Concavity and geometric descriptors				
Johns et al. [25]	Community adults (890)	Central and peripheral concavity	Respiratory limitation/spirometric obstruction	r with FEV_1_/FVC and FEF_25–75_%: −0.70 to −0.79; ICC ≈ 0.96
Dominelli et al. [27]	Mild COPD vs. healthy controls (34)	Slope-ratio	Mild COPD discrimination	Altered SR-volume pattern despite preserved FEV_1_/FVC
Wang et al. [28]	Population and clinical cohorts (18,938)	β angle; central/peripheral concavity	Spirometric pattern classification	β angle AUC 0.97; cutoff 163°; accuracy 0.919
Mochizuki et al. [13]	Stable obstructive lung disease (133)	Obstructive Index	Emphysema on CT (LAV%)	AUC 0.819; cutoff 4.38; ρ with LAV% = 0.56
Tan et al. [26]	Population cohort, 8-year follow-up (852)	Central concavity	Incident COPD	AUC 0.76; Se 0.70; Sp 0.79; NPV 0.99
Bhatt et al. [29]	Smokers/former smokers (8390)	Lung size-adjusted Peak Index	Morbidity, mortality, CT structural disease, FEV_1_ decline	r(FEV_1_/FVC) = −0.73; r(FEV_1_%) = −0.67; ICC 0.923
Kuo et al. [41]	Mechanically ventilated ICU patients (33)	Degree of convexity	COPD vs. non-COPD	AUC 0.70; Se 0.778; Sp 0.533
Expiratory slope and flow-decay metrics				
Moreno-Giraldo et al. [16]	Chronic respiratory symptoms (765)	PEF–FVC slope	COPD by post-BD FEV_1_/FVC < 0.70	Cutoff > −1.76; AUC 0.98; Se 0.94; Sp 0.94; ICC 0.83
Oh et al. [15]	Pulmonary function laboratory (125)	Flow decay	Reversible obstruction; obstruction with hyperinflation/air trapping	Cutoff 0.802 L^−1^; AUC 0.95–0.98; CV 9.8 ± 3.4%
Bodduluri et al. [30]	Population cohort and at-risk smokers (8301)	Spirometric time constant	Exacerbations, emphysema, mortality	Cutoff 0.76 s; associated with GOLD progression, emphysema, small-airway disease
Weber et al. [31]	Occupational surveillance cohort (10,810)	Longitudinal low-FVC transition pattern	Transition to low-FVC pattern	Low-FVC defined by FVC < LLN and FEV_1_/FVC > LLN; r(FEV_1_, FVC) = 0.95
Area-derived metrics				
Zapletal et al. [32]	Pediatric challenge/reversibility testing (142 challenge tests; 110 bronchodilation tests; 9 CF cases)	Flow–volume curve area (AEX)	Bronchial hyperresponsiveness/bronchodilator response	≥20% AEX change; sensitivity 100% for BHR vs. 36% with FEV_1_
Ioachimescu et al. [42]	Pulmonary function laboratory (15,308)	Actual AEX	Normal, obstructive, restrictive, mixed patterns	AUROC 0.97–0.99; predicted IC, IC/TLC, RV/TLC
Ioachimescu et al. [20]	Pulmonary function laboratory (15,308)	AEX4	Ventilatory pattern classification	AUROC 0.96–0.99; correlation with actual AEX 0.95–0.99
Desyatskova et al. [33]	Patients with respiratory disease (540)	AEX-FV	Bronchial obstruction	Cutoff 74–85%; Se 0.824–0.916; Sp 0.800–0.933
Lee et al. [34]	Stable COPD (61)	Obstruction area normalized by triangle area	Hyperinflation/exercise capacity	Ao with RV/TLC: r = 0.674; Ao/AR and Ao/PEF associated with 6MWD
Das et al. [35]	COPD (319)	AreaFE%	Severe hyperinflation (RV/TLC > 60%; IC/TLC < 25%)	AUC 0.91; accuracy 0.80; r with RV/TLC −0.78 and IC/TLC 0.80
Satici et al. [36]	COPD with severe dyspnea (81)	AreaFE%	Severe exacerbation	Cutoff 0.17; AUC 0.88; Se 0.818; Sp 0.828; NPV 0.96
Li et al. [18]	Hospital spirometry database (500)	AUC3/AT3	Airflow limitation by FEV_1_/FVC	r = 0.88; Se 0.865; Sp 0.859; accuracy 0.863
Wang et al. [37]	Primary care (767)	FEV_1_/FEV_6_	COPD vs. FEV_1_/FVC < 0.70	Cutoff 0.72; AUC 0.98; Se 0.969; Sp 0.988
Moreno-Giraldo et al. [16]	Chronic respiratory symptoms (765)	Area-derived metrics	COPD by post-BD FEV_1_/FVC < 0.70	ICC 0.93–0.97; AUC 0.69–0.81

AEX, area under the expiratory curve; AEX-FV, area under the flow–volume curve; Ao, obstruction area; AreaFE%, expiratory flow–volume curve area expressed as a percentage of a reference value; AUC/AUROC, area under the receiver operating characteristic curve; BD, bronchodilator; BHR, bronchial hyperresponsiveness; COPD, chronic obstructive pulmonary disease; CT, computed tomography; CV, coefficient of variation; FEF_25–75_%, forced expiratory flow between 25% and 75% of FVC; FEV_1_, forced expiratory volume in one second; FEV_6_, forced expiratory volume in six seconds; FVC, forced vital capacity; ICC, intraclass correlation coefficient; IC, inspiratory capacity; LAV%, low-attenuation volume percentage; LLN, lower limit of normal; NPV, negative predictive value; PEF, peak expiratory flow; RV/TLC, residual volume/total lung capacity; Se, sensitivity; Sp, specificity.

## Data Availability

No new data were created or analyzed in this study. Data sharing is not applicable to this article.

## Supplementary Materials

The following supporting information can be downloaded at: https://www.mdpi.com/article/10.3390/arm94050061/s1, Table S1. PRISMA Extension for Scoping Reviews (PRISMA-ScR) 2018 Checklist [24]; Table S2: Search Strategies.

## References

[1] Vu S.P., Veit K., Sadikot R.T. (2025). Molecular Approaches to Treating Chronic Obstructive Pulmonary Disease: Current Perspectives and Future Directions. Int. J. Mol. Sci..

[2] Wang Z., Lin J., Liang L., Huang F., Yao X., Peng K., Gao Y., Zheng J. (2025). Global, regional, and national burden of chronic obstructive pulmonary disease and its attributable risk factors from 1990 to 2021: An analysis for the Global Burden of Disease Study 2021. Respir. Res..

[3] Al Wachami N., Guennouni M., Iderdar Y., Boumendil K., Arraji M., Mourajid Y., Bouchachi F.Z., Barkaoui M., Louerdi M.L., Hilali A. (2024). Estimating the global prevalence of chronic obstructive pulmonary disease (COPD): A systematic review and meta-analysis. BMC Public Health.

[4] Boers E., Barrett M., Su J.G., Benjafield A.V., Sinha S., Kaye L., Zar H.J., Vuong V., Tellez D., Gondalia R. (2023). Global Burden of Chronic Obstructive Pulmonary Disease Through 2050. JAMA Netw. Open.

[5] Chen S., Kuhn M., Prettner K., Yu F., Yang T., Bärnighausen T., Bloom D.E., Wang C. (2023). The global economic burden of chronic obstructive pulmonary disease for 204 countries and territories in 2020–50: A health-augmented macroeconomic modelling study. Lancet Glob. Health.

[6] Caballero A., Torres-Duque C.A., Jaramillo C., Bolívar F., Sanabria F., Osorio P., Orduz C., Guevara D.P., Maldonado D. (2008). Prevalence of COPD in Five Colombian Cities Situated at Low, Medium, and High Altitude (PREPOCOL Study). Chest.

[7] GOLD Science Committee Members (2024–2025) (2025). Global Strategy for Prevention, Diagnosis and Management of COPD: 2025 Report.

[8] Maldonado-Franco A., Giraldo-Cadavid L.F., Tuta-Quintero E., Bastidas Goyes A.R., Botero-Rosas D.A. (2023). The Challenges of Spirometric Diagnosis of COPD. Can. Respir. J..

[9] Bhatt S.P., Bhakta N.R., Wilson C.G., Cooper C.B., Barjaktarevic I., Bodduluri S., Kim Y.I., Eberlein M., Woodruff P.G., Sciurba F.C. (2018). New Spirometry Indices for Detecting Mild Airflow Obstruction. Sci. Rep..

[10] Nozoe M., Mase K., Murakami S., Okada M., Ogino T., Matsushita K., Takashima S., Yamamoto N., Fukuda Y., Domen K. (2013). Relationship between spontaneous expiratory flow-volume curve pattern and air-flow obstruction in elderly COPD patients. Respir. Care.

[11] Zhou R., Wang H., Zhang Y., Mai J., Yang L. (2025). Small airway disease as a key factor in COPD: New perspectives and insights. Front. Med..

[12] Chukowry P.S., Spittle D.A., Turner A.M. (2021). Small Airways Disease, Biomarkers and COPD: Where are We?. Int. J. Chronic Obstr. Pulm. Dis..

[13] Mochizuki F., Iijima H., Watanabe A., Tanabe N., Sato S., Shiigai M., Fujiwara K., Shimada T., Ishikawa H., Kanazawa J. (2019). The Concavity of the Maximal Expiratory Flow–Volume Curve Reflects the Extent of Emphysema in Obstructive Lung Diseases. Sci. Rep..

[14] Verstraete K., Das N., Gyselinck I., Topalovic M., Troosters T., Crapo J.D., Silverman E.K., Make B.J., Regan E.A., Jensen R. (2023). Principal component analysis of flow-volume curves in COPDGene to link spirometry with phenotypes of COPD. Respir. Res..

[15] Oh A., Morris T.A., Yoshii I.T., Morris T.A. (2017). Flow Decay: A Novel Spirometric Index to Quantify Dynamic Airway Resistance. Respir. Care.

[16] Giraldo A.M.M., Cadavid L.F.G., Rosas D.B., Quintero E.T., Maldonado-Franco A., Murcia H.C.A., Suárez C.E.A., Cely L.M.M., Bastidas A.R. (2023). Comparison of New Spirometry Measures to Diagnose COPD. Respir. Care.

[17] Bhatt S.P., Soler X., Wang X., Murray S., Anzueto A.R., Beaty T.H., Boriek A.M., Casaburi R., Criner G.J., Diaz A.A. (2016). Association between Functional Small Airway Disease and FEV1 Decline in Chronic Obstructive Pulmonary Disease. Am. J. Respir. Crit. Care Med..

[18] Li H., Liu C., Zhang Y., Xiao W. (2017). The Concave Shape of the Forced Expiratory Flow-Volume Curve in 3 Seconds Is a Practical Surrogate of FEV1/FVC for the Diagnosis of Airway Limitation in Inadequate Spirometry. Respir. Care.

[19] Ioachimescu O.C., Ramos J.A., Hoffman M., Stoller J.K. (2021). Area under the expiratory flow-volume curve: Predicted values by regression and deep learning methods and recommendations for clinical practice. BMJ Open Resp. Res..

[20] Ioachimescu O.C., Stoller J.K. (2020). An Alternative Spirometric Measurement. Area under the Expiratory Flow–Volume Curve. Ann. Am. Thorac. Soc..

[21] Arksey H., O’Malley L. (2005). Scoping studies: Towards a methodological framework. Int. J. Soc. Res. Methodol..

[22] Levac D., Colquhoun H., O’Brien K.K. (2010). Scoping studies: Advancing the methodology. Implement. Sci..

[23] Peters M.D.J., Marnie C., Tricco A.C., Pollock D., Munn Z., Alexander L., McInerney P., Godfrey C.M., Khalil H. (2020). Updated methodological guidance for the conduct of scoping reviews. JBI Evid. Synth..

[24] Tricco A.C., Lillie E., Zarin W., O’Brien K.K., Colquhoun H., Levac D., Moher D., Peters M.D.J., Horsley T., Weeks L. (2018). PRISMA Extension for Scoping Reviews (PRISMA-ScR): Checklist and Explanation. Ann. Intern. Med..

[25] Johns D.P., Das A., Toelle B.G., Abramson M.J., Marks G.B., Wood-Baker R., Walters E.H. (2017). Improved spirometric detection of small airway narrowing: Concavity in the expiratory flow–volume curve in people aged over 40 years. Int. J. Chronic Obstr. Pulm. Dis..

[26] Tan D.J., Johns D.P., Lodge C.J., Bui D.S., Vicendese D., Hamilton G.S., Han M.K., Agusti A., Abramson M.J., Perret J.L. (2026). Concavity of the maximal expiratory flow–volume curve, and incidence of COPD and respiratory symptoms: A population-based cohort study. ERJ Open Res..

[27] Dominelli P.B., Foster G.E., Guenette J.A., Haverkamp H.C., Eves N.D., Dominelli G.S., Henderson W.R., O’Donnell D.E., Sheel A.W. (2015). Quantifying the shape of the maximal expiratory flow–volume curve in mild COPD. Respir. Physiol. Neurobiol..

[28] Wang Z., Liang L., Huang F., Peng K., Lin J., Gao Y., Zheng J. (2025). The Characteristics of the Concavity of Descending Limb of Maximal Expiratory Flow-Volume Curves Generated by Spirometry. Lung.

[29] Bhatt S.P., Bodduluri S., Raghav V., Bhakta N.R., Wilson C.G., Kim Y.I., Eberlein M., Sciurba F.C., Han M.K., Dransfield M.T. (2019). The Peak Index: Spirometry Metric for Airflow Obstruction Severity and Heterogeneity. Ann. Am. Thorac. Soc..

[30] Bodduluri S., Nakhmani A., Bhatt S.P. Spirometric time constant for detection of early and mild airflow obstruction. Proceedings of the American Thoracic Society International Conference.

[31] Weber J., Zhang Z., Doucette J.T., Sood A., Celedón J.C., De La Hoz R.E. (2025). Spirometric pattern transitions in a World Trade Center occupational cohort on longitudinal surveillance. Respir. Med..

[32] Zapletal A., Hladíková M., Chalupová J., Svobodová T., Vávrová V. (2008). Area under the Maximum Expiratory Flow-Volume Curve—A Sensitive Parameter in the Evaluation of Airway Patency. Respiration.

[33] Desyatskova E., Grechenko V., Soboleva V. (2024). Analysis of the possibilities of the flow-volume curve assessment by the changes in its shape in patients with obstructive airway diseases. Bull. Russ. State Med. Univ..

[34] Lee J., Lee C.T., Lee J.H., Cho Y.J., Park J.S., Oh Y.M., Lee S.D., Yoon H.I., KOLD Study Group (2016). Graphic analysis of flow-volume curves: A pilot study. BMC Pulm. Med..

[35] Das N., Topalovic M., Aerts J.M., Janssens W. (2019). Area under the forced expiratory flow-volume loop in spirometry indicates severe hyperinflation in COPD patients. Int. J. Chronic Obstr. Pulm. Dis..

[36] Satici C., Demirkol M.A., Arpinar Yigitbas B., Erinc A., Kosar A.F. (2021). Area under flow-volume loop may predict severe exacerbation in COPD patients with high grade of dyspnea. Respir. Physiol. Neurobiol..

[37] Wang S., Gong W., Tian Y., Zhou J. (2016). FEV_1_ /FEV_6_ in Primary Care Is a Reliable and Easy Method for the Diagnosis of COPD. Respir. Care.

[38] Maldonado-Franco A., Giraldo-Cadavid L.F., Tuta-Quintero E., Cagy M., Bastidas Goyes A.R., Botero-Rosas D.A. (2024). Curve-Modelling and Machine Learning for a Better COPD Diagnosis. Int. J. Chronic Obstr. Pulm. Dis..

[39] Hebbink R.H.J., Elshof J., Wijkstra P.J., Duiverman M.L., Hagmeijer R. (2024). On the relation between tidal and forced spirometry. Med. Eng. Phys..

[40] Gadgil S., Galanter J.M., Negahdar M. (2025). Leveraging raw spirometry data and deep learning for improved COPD diagnosis and risk prediction. Am. J. Respir. Crit. Care Med..

[41] Kuo Y.L., Kao J.H., Chen Y.W., Wang J.H., Lu C.C., Ko H.K. (2021). Degree of convexity calculated from expiratory flow-volume curves for identifying airway obstruction in nonsedated and nonparalyzed ventilated patients. Respir. Physiol. Neurobiol..

[42] Ioachimescu O.C., Stoller J.K. (2020). Area under the expiratory flow-volume curve (AEX): Actual versus approximated values. J. Investig. Med..

[43] Dawson S.V., Elliott E.A. (1977). Wave-speed limitation on expiratory flow-a unifying concept. J. Appl. Physiol..

[44] Alowiwi H., Watson S., Jetmalani K., Thamrin C., Johns D.P., Walters E.H., King G.G. (2022). Relationship between concavity of the flow-volume loop and small airway measures in smokers with normal spirometry. BMC Pulm. Med..

[45] Hogg J.C., Chu F., Utokaparch S., Woods R., Elliott W.M., Buzatu L., Cherniack R.M., Rogers R.M., Sciurba F.C., Coxson H.O. (2004). The Nature of Small-Airway Obstruction in Chronic Obstructive Pulmonary Disease. N. Engl. J. Med..

[46] Sarkar M., Madabhavi I.V., Mehta S., Mohanty S. (2022). Use of flow volume curve to evaluate large airway obstruction. Monaldi Arch. Chest Dis..

[47] Abboud S., Barnea O., Guber A., Narkiss N., Bruderman I. (1995). Maximum expiratory flow-volume curve: Mathematical model and experimental results. Med. Eng. Phys..

