# Supplementary material for: Morphological Analysis of the Flow–Volume Curve for Identifying Fixed Airflow Obstruction: A Scoping Review
Source: Adv Respir Med. 2026 Aug 29;94(5):61. doi: 10.3390/arm94050061 (PMC13600186; doi:10.3390/arm94050061)
Supplement: Supplementary file 1 [file arm-94-00061-s001.zip › arm-4464170-supplementary.pdf]

Supplementary Table S1. PRISMA Extension for Scoping Reviews (PRISMA-ScR) 2018 Checklist[1]

Section/topic	#	PRISMA-ScR Checklist item	Reported on page #
**TITLE**			
Title	1	Identify the report as a scoping review.	1
**ABSTRACT**			
Structured summary	2	Provide a structured summary including, as applicable: background; objectives; data sources; study eligibility criteria, participants, and interventions; study synthesis methods; results; limitations; conclusions and implications of key findings.	1
**INTRODUCTION**			
Rationale	3	Describe the rationale for the review in the context of what is already known. Explain why the review question(s)/objective(s) lend themselves to a scoping review approach.	2
Objectives	4	Provide an explicit statement of the question(s) and objective(s) being addressed with reference to their key elements (e.g., population or participants, concepts and context), or other relevant key elements used to conceptualize the review question(s) and/or objective(s)).	2
**METHODS**			
Protocol and registration	5	Indicate if a review protocol exists, if and where it can be accessed (e.g., Web address), and, if available, provide registration information including registration number.	3
Eligibility criteria	6	Specify the characteristics of the sources of evidence (e.g., years considered, language, publication status) used as criteria for eligibility, and provide a rationale.	3
Information sources	7	Describe all information sources (e.g., databases with dates of coverage, contact with study authors to identify additional sources) in the search and date last searched.	3
Search	8	Present full electronic search strategy for at least one database, including any limits used, such that it could be repeated.	Supplementary file
Selection of sources of evidence	9	State the process for selecting studies (i.e., screening, eligibility) included in the scoping review.	4
Data charting process	10	Describe the methods of charting data from the included sources of evidence (e.g. piloted forms; forms that have been tested by the team before their use, whether data charting was done independently, in duplicate) and any processes for obtaining and confirming data from investigators.	4
Data items	11	List and define all variables for which data were sought and any assumptions and simplifications made.	5

Section/topic	#	PRISMA-ScR Checklist item	Reported on page #
Critical appraisal of individual sources of evidence	12	**If done,** provide a rationale for conducting a critical appraisal of included sources of evidence; describe the methods used and how this information was used in any data synthesis (if appropriate).	Not applicable
Summary measures	13	Not applicable for scoping reviews.	Not applicable
Synthesis of results	14	Describe the methods of handling and summarizing the data that were charted.	4
Risk of bias across studies	15	Not applicable for scoping reviews.	Not applicable
Additional analyses	16	Not applicable for scoping reviews.	Not applicable
**RESULTS**			
Selection of sources of evidence	17	Give numbers of studies screened, assessed for eligibility, and included in the review, with reasons for exclusions at each stage, ideally using a flow diagram.	Figure 1<br>5-8
Characteristics of sources of evidence	18	For each source of evidence, present characteristics for which data were charted and provide the citations.	Table 1 and table 2<br>5-8
Critical appraisal within sources of evidence	19	If done, present data on critical appraisal of included sources of evidence (see item 12).	Not applicable
Results of individual sources of evidence	20	For each included source of evidence, present the relevant data that were charted that relate to the review question(s) and objective(s).	Table 1 and table 2<br>5-8
Synthesis of results	21	Summarize and/or present the charting results as they relate to the review question(s) and objective(s).	Table 1 and<br>5-8
Risk of bias across studies	22	Not applicable for scoping reviews.	NA
Additional analysis	23	Not applicable for scoping reviews.	NA
**DISCUSSION**			
Summary of evidence	24	Summarize the main results (including an overview of concepts, themes, and types of evidence available), explain how they relate to the review question(s) and objectives, and consider the relevance to key groups	9-10
Limitations	25	Discuss the limitations of the scoping review process.	10

Section/topic	#	PRISMA-ScR Checklist item	Reported on page #
Conclusions	26	Provide a general interpretation of the results with respect to the review question(s) and objective(s), as well as potential implications and/or next steps.	11
**FUNDING**			
Funding	27	Describe sources of funding for the included sources of evidence, as well as sources of funding for the scoping review. Describe the role of the funders of the scoping review.	11

Tricco AC, Lillie E, Zarin W, et al. PRISMA Extension for Scoping Reviews (PRISMA-ScR): Checklist and Explanation. *Ann Intern Med* 2018;169(7):467-73.

Supplementary Table S2. Search Strategies

**Pubmed**<br>20260101_Revision_morfologia_PRIMS_Busqueda_Embase_2000_2025<br>("Risk Factors"[Mesh] OR "Smoking"[Mesh] OR "Tobacco Use"[Mesh] OR "Air Pollution"[Mesh] OR "Environmental Exposure"[Mesh] OR "Environmental Pollutants"[Mesh] OR "Biomass"[Mesh] OR "Aging"[Mesh] OR smoker*[tiab] OR smoking[tiab] OR biomass[tiab] OR "biomass smoke"[tiab] OR pollution[tiab] OR pollutants[tiab] OR "household air pollution"[tiab] OR aging[tiab] OR age[tiab]) AND ("flow-volume"[tiab] OR "flow volume"[tiab] OR "flow-volume loop"[tiab] OR "flow volume loop"[tiab] OR "flow-volume curve"[tiab] OR "flow volume curve"[tiab] OR "expiratory flow-volume"[tiab] OR "expiratory flow volume"[tiab] OR "maximal expiratory flow-volume"[tiab] OR "maximal expiratory flow volume"[tiab] OR MEFV[tiab] OR MEFVC[tiab] OR "spirometric curve"[tiab] OR "spirometric curves"[tiab] OR "expiratory limb"[tiab] OR "descending limb"[tiab] OR morpholog*[tiab] OR morphometr*[tiab] OR shape[tiab] OR contour[tiab] OR "shape analysis"[tiab] OR "shape descriptor"[tiab] OR "shape descriptors"[tiab] OR pattern[tiab] OR "pattern analysis"[tiab] OR scooped[tiab] OR "scooped-out"[tiab] OR scooping[tiab] OR concav*[tiab] OR curvature[tiab] OR curvatur*[tiab] OR "concavity index"[tiab] OR "shape index"[tiab] OR "beta angle"[tiab] OR "angle beta"[tiab] OR "slope ratio"[tiab] OR "slope-ratio"[tiab] OR "obstructive index"[tiab] OR "peak index"[tiab] OR "angle of collapse"[tiab] OR "airway collapse"[tiab] OR AEX[tiab] OR "area under"[tiab] OR AreaFE[tiab] OR "areaFE"[tiab] OR "rectangular area ratio"[tiab] OR RAR[tiab] OR (expiratory[tiab] AND flow[tiab] AND volume[tiab] AND area[tiab]) OR slope*[tiab] OR "flow decay"[tiab] OR "time constant"[tiab] OR "time constants"[tiab] OR "mean transit time"[tiab] OR MTT[tiab] OR "transit time"[tiab] OR "transit time analysis"[tiab] OR "curve fitting"[tiab] OR "curve modeling"[tiab] OR polynomial*[tiab] OR spline*[tiab] OR exponential[tiab] OR "exponential model"[tiab] OR "mathematical model"[tiab] OR modeling[tiab] OR modelling[tiab] OR "principal component"[tiab] OR "principal components"[tiab] OR "principal component analysis"[tiab] OR PCA[tiab] OR "feature extraction"[tiab] OR "pattern recognition"[tiab] OR "machine learning"[tiab] OR "deep learning"[tiab] OR "artificial intelligence"[tiab] OR "neural network"[tiab] OR "neural networks"[tiab] OR "artificial neural network"[tiab] OR "artificial neural networks"[tiab]) AND ("Pulmonary Disease, Chronic Obstructive"[Mesh] OR "Lung Diseases, Obstructive"[Mesh] OR COPD[tiab] OR "chronic obstructive pulmonary disease"[tiab] OR "airflow obstruction"[tiab] OR "airflow limitation"[tiab] OR "obstructive lung disease"[tiab]) Filters: Humans, from 2000 - 2025<br>Total: 5535.
**Embase**<br>20260124_Revision_morfologia_PRIMS_Busqueda_Embase_2000_2025<br>('smoking'/exp OR 'tobacco use'/exp OR 'air pollution'/exp OR 'biomass'/exp OR smoker*:ti,ab,kw OR smoking:ti,ab,kw OR biomass:ti,ab,kw OR 'biomass smoke':ti,ab,kw OR 'household air pollution':ti,ab,kw) AND ('flow-volume loop':ti,ab,kw OR 'flow volume loop':ti,ab,kw OR 'flow-volume curve':ti,ab,kw OR 'flow volume curve':ti,ab,kw OR 'expiratory flow-volume':ti,ab,kw OR 'expiratory flow volume':ti,ab,kw OR 'maximal expiratory flow-volume':ti,ab,kw OR 'maximal expiratory flow volume':ti,ab,kw OR mefv:ti,ab,kw OR mefvc:ti,ab,kw OR 'expiratory limb':ti,ab,kw

OR 'descending limb':ti,ab,kw OR concav*:ti,ab,kw OR curvature:ti,ab,kw OR curvatur*:ti,ab,kw OR 'concavity index':ti,ab,kw OR 'shape index':ti,ab,kw OR 'beta angle':ti,ab,kw OR 'angle beta':ti,ab,kw OR 'slope ratio':ti,ab,kw OR 'slope-ratio':ti,ab,kw OR 'obstructive index':ti,ab,kw OR 'peak index':ti,ab,kw OR aex:ti,ab,kw OR areafe:ti,ab,kw OR 'rectangular area ratio':ti,ab,kw OR rar:ti,ab,kw OR (expiratory:ti,ab,kw AND flow:ti,ab,kw AND volume:ti,ab,kw AND area:ti,ab,kw) OR 'flow decay':ti,ab,kw OR 'time constant':ti,ab,kw OR 'time constants':ti,ab,kw OR 'mean transit time':ti,ab,kw OR mtt:ti,ab,kw OR 'transit time analysis':ti,ab,kw OR 'curve fitting':ti,ab,kw OR polynomial*:ti,ab,kw OR spline*:ti,ab,kw OR exponential*:ti,ab,kw OR 'exponential model':ti,ab,kw OR 'principal component analysis':ti,ab,kw OR pca:ti,ab,kw) AND ('chronic obstructive lung disease'/exp OR 'chronic obstructive pulmonary disease'/exp OR 'obstructive lung disease'/exp OR copd:ti,ab,kw OR 'chronic obstructive pulmonary disease':ti,ab,kw OR 'airflow obstruction':ti,ab,kw OR 'airflow limitation':ti,ab,kw) AND [humans]/lim AND [2000-2025]/py<br>Total: 951.
**SCOPUS**<br>20260124_Revision_morfologia_PRIMS_Busqueda_Scopus_2000_2025<br>TITLE-ABS-KEY ((COPD OR "chronic obstructive pulmonary disease" OR "obstructive lung disease*" OR "airflow obstruction" OR "airflow limitation") AND (smoking OR smoker* OR "tobacco use" OR "air pollution" OR "environmental exposure" OR "environmental pollutant*" OR biomass OR "biomass smoke" OR "household air pollution" OR aging OR age) AND (("flow-volume loop" OR "flow-volume curve" OR "flow volume loop" OR "flow volume curve" OR "expiratory flow-volume" OR "maximal expiratory flow-volume" OR MEFV OR MEFVC OR "spirometric curve*" OR ("spirometry" AND ("flow-volume" OR "flow volume"))) OR (shape OR contour OR "shape analysis" OR "pattern analysis" OR modeling OR modelling OR "curve modeling" OR "curve fitting" OR "mathematical model*" OR morpholog* OR morphometr*))) AND (LIMIT-TO (EXACTKEYWORD , "Human"))<br>Total: 2327
**Open Aire**<br>20260124_Revision_morfologia_PRIMS_Busqueda_OpenAire_2000_2025<br>(("chronic obstructive pulmonary disease" OR COPD OR "obstructive lung disease" OR "airflow obstruction") AND ("flow-volume" OR "flow volume" OR "flow-volume curve" OR "flow-volume loop" OR "expiratory flow" OR spirometry) AND (morphology OR morphometric OR concavity OR curvature OR shape OR slope OR area OR "curve analysis" OR "curve fitting" OR "mathematical model" OR "pattern recognition"))<br>Total: 1099
**Web of Science**<br>20260124_Revision_morfologia_PRIMS_Busqueda_Web_of_Sience_2000_2025<br>TS=(("chronic obstructive pulmonary disease" OR COPD OR "obstructive lung disease" OR "airflow obstruction") AND ("flow-volume" OR "flow volume" OR "expiratory flow" OR spirometry OR "lung function") AND (morphology OR morphometric OR concavity OR curvature OR shape OR slope OR area OR AEX OR "curve analysis" OR "curve fitting" OR "mathematical model" OR "pattern recognition" OR "machine learning"))

NOT TS=(animal* OR mice OR mouse OR rat OR rats OR murine OR canine OR porcine OR sheep OR bovine OR rabbit* OR dog* OR cat*)<br>Total: 1862.
**Lilacs:**<br>20260124_Revision_morfologia_PRIMS_Busqueda_Lilacs_2000_2025<br>((mh:“Tabaquismo” OR mh:“Uso de Tabaco” OR mh:“Contaminación del Aire” OR mh:“Exposición Ambiental” OR mh:“Contaminantes Ambientales” OR mh:“Biomasa” OR mh:“Envejecimiento” OR tw:smoker* OR tw:smoking OR tw:“biomass smoke” OR tw:biomass OR tw:“household air pollution” OR tw:pollution OR tw:aging) AND (tw:“flow-volume curve” OR tw:“flow volume curve” OR tw:“flow-volume loop” OR tw:“expiratory flow-volume” OR tw:“maximal expiratory flow-volume” OR tw:“expiratory limb” OR tw:concav* OR tw:curvature OR tw:“beta angle” OR tw:“slope ratio” OR tw:“slope-ratio” OR tw:AEX OR tw:“area under” OR tw:“rectangular area ratio” OR tw:RAR OR tw:“airway collapse” OR tw:“curve fitting” OR tw:“curve modeling” OR tw:“mathematical model” OR tw:polynomial* OR tw:spline* OR tw:“principal component analysis” OR tw:PCA OR tw:“feature extraction” OR tw:“pattern recognition” OR tw:“machine learning” OR tw:“deep learning” OR tw:“artificial intelligence” OR tw:“neural network*”) AND (mh:“Enfermedad Pulmonar Obstructiva Crónica” OR mh:“Enfermedades Pulmonares Obstructivas” OR tw:COPD OR tw:“chronic obstructive pulmonary disease” OR tw:“airflow obstruction” OR tw:“airflow limitation” OR tw:“obstructive lung disease”))<br>Total: 934.
**IEEE:**<br>20260124_Revision_morfologia_PRIMS_Busqueda_IEEE_2000_2025<br>("Full Text & Metadata":chronic obstructive pulmonary disease OR "Full Text & Metadata":COPD) AND ("Full Text & Metadata":spirometry OR "Full Text & Metadata":flow-volume OR "Full Text & Metadata":flow volume OR "Full Text & Metadata":spirometric curve) AND ("Full Text & Metadata":smoking OR "Full Text & Metadata":air pollution OR "Full Text & Metadata":aging OR "Full Text & Metadata":biomass smoke) AND ("Full Text & Metadata":curve analysis OR "Full Text & Metadata":morphology OR "Full Text & Metadata":concavity OR "Full Text & Metadata":shape OR "Full Text & Metadata":machine learning)<br>Total: 869.
